# Is the Standard Artificial Urinary Sphincter AMS 800 Still a Treatment Option for the Irradiated Male Patient Presenting with a Devastated Bladder Outlet?

**DOI:** 10.3390/jcm12124002

**Published:** 2023-06-12

**Authors:** Fabian Queissert, Tanja Huesch, Alexander Kretschmer, Ruth Kirschner-Hermanns, Tobias Pottek, Roberto Olianas, Alexander Friedl, Roland Homberg, Jesco Pfitzenmaier, Carsten M. Naumann, Joanne Nyarangi-Dix, Torben Hofmann, Achim Rose, Christian Weidemann, Carola Wotzka, Wilhelm Hübner, Hagen Loertzer, Rudi Abdunnur, Markus Grabbert, Ralf Anding, Ricarda M. Bauer, Axel Haferkamp, Andres J. Schrader

**Affiliations:** 1 Department of Urology and Pediatric Urology, University Hospital Münster, 48149 Münster, Germany; 2 Department of Urology, University Medical Center of Johannes Gutenberg University, 55131 Mainz, Germany; 3 Department of Urology, Ludwig-Maximilians University, Campus Großhadern, 80539 Munich, Germany; 4 Department of Urology, University Hospital Bonn, 53127 Bonn, Germany; 5 Department of Urology, Vivantes Hospital Am Urban, 10117 Berlin, Germany; 6 Department of Urology, Hospital Lüneburg, 21339 Lüneburg, Germany; 7 Department of Urology, Göttlicher Heiland Vienna, 1170 Vienna, Austria; 8 Department of Urology and Pediatric Urology, St. Barbara Hospital Hamm, 59075 Hamm, Germany; 9 Department of Urology, Evangelic Hospital Bethel, 42240 Bielefeld, Germany; 10 Department of Urology and Pediatric Urology, St. Elisabeth Hospital, 56564 Neuwied, Germany; 11 Department of Urology and Pediatric Urology, University Hospital Heidelberg, 69120 Heidelberg, Germany; 12 Department of Urology, Diakonie Hospital Schwaebisch Hall, 74523 Schwaebisch Hall, Germany; 13 Department of Urology and Pediatric Urology, Helios Hospital Duisburg, 47166 Duisburg, Germany; 14 Department of Urology and Pediatric Urology, Catholic Hospital St. Johann Nepomuk, 99097 Erfurt, Germany; 15 Department of Urology, Diakonie Hospital Stuttgart, 70176 Stuttgart, Germany; 16 Department of Urology, Hospital Weinviertel Korneuburg, 2100 Korneuburg, Austria; 17 Department of Urology and Pediatric Urology, Westpfalz Medical Center, 67655 Kaiserslautern, Germany; 18 Department of Urology, Helios Hospital Schwelm, 58332 Schwelm, Germany; 19 Department of Urology, University Hospital Freiburg, 79106 Freiburg, Germany; 20 Department of Urology, University Hospital Basel, 4031 Basel, Switzerland

**Keywords:** devastated bladder outlet, AMS 800, artificial urinary sphincter, male stress incontinence, prostate radiotherapy, bladder neck stenosis

## Abstract

Background: Circular urethral compression with an artificial sphincter allows control of voiding, even in patients with severe stress urinary incontinence, but it heightens the risk of urethral atrophy and erosion. This study of one of the largest populations of patients treated with radiotherapy investigates the additive effect of the post-radiogenic stricture of the membranous urethra/bladder neck on AMS 800 artificial urinary sphincter outcomes. Methods: In a retrospective multicenter cohort study, we analyzed patients fitted with an AMS 800, comparing those who had received radiotherapy with patients presenting a devastated bladder outlet (stricture of the membranous urethra/bladder neck). We determined the correlation between these groups of patients using both univariate and stepwise adjusted multivariate regression. The revision-free interval was estimated by a Kaplan–Meier plot and compared by applying the log-rank test. A *p* value below 0.05 was considered statistically significant. Results: Of the 123 irradiated patients we identified, 62 (50.4%) had undergone at least one prior desobstruction for bladder-neck/urethra stenosis. After a mean follow-up of 21 months, the latter tended to achieve social continence less frequently (25.7% vs. 35%; *p* = 0.08). Revision was required significantly more often for this group (43.1% vs. 26.3%; *p* = 0.05) due to urethral erosion in 18 of 25 cases. A stenosis recurred in five cases; desobstruction was performed in two cases, leading to erosion in both. Multivariate analysis revealed a significantly higher risk of revision when recurrent stenosis necessitated at least two prior desobstructions (HR 2.8; *p* = 0.003). Conclusions: A devastated bladder outlet is associated with a lower proportion of men with social continence and a significantly higher need for revision compared with irradiated patients without a history of urethral stenosis. Alternative surgical procedures should be discussed beforehand, especially in cases of recurrent urethral stenosis.

## 1. Introduction

The prevalence of stress urinary incontinence (SUI) after radical prostatectomy (RPx) ranges from 2% to 20% of patients, depending on the time of acquisition and definition [1]. However, prevalence and expression increase in cases where radiotherapy (RT) was performed. In particular, implementation before RPx (primary RT, 50% of cases) and soon after RPx (adjuvant RT, up to 20% of cases) is associated with a marked expression and accumulation of SUI [2,3]. Histopathological examination of patients with RT demonstrates specific histologic characteristics, including vascular loss and increased scarring (collagen density, organization), in the membranous urethra/bladder neck (mU/BN) [4]. In addition to worsening the continence situation, the said changes may also lead to a stricture in the area of the mU/BN. The incidence of relevant stenosis depends on the modality used, varying from 2% for external radiotherapy to 32% for brachytherapy [4]. Notably, in contrast to postoperative anastomotic stenosis, post-radiogenic stricture occurs with a time delay and with a rising incidence over the years [5,6]. If both stress incontinence and stricture of the bladder neck or membranous urethra are present simultaneously in irradiated patients, the term “devastated bladder outlet” (dBO) applies [7], leading to considerable impairment of daily life. If the stenosis dominates with frequent recurrences, a perineal urethroplasty can be performed. However, this is associated with a worse outcome compared to non-irradiated patients [8,9,10]. Various forms of supravesical urinary diversion are discussed as a last resort [7,8]. On the other hand, in the absence of rapid restenosis and with persisting stress incontinence, continence surgery may be attempted. In severe stress incontinence, an AUS is generally preferred, with erosion and urethral atrophy occurring more frequently in irradiated patients [10]. This was confirmed in an initial analysis of our cohort [11], with a high ASA score and a status post urethral stricture additionally identified as risk factors. Ultimately, however, the outcome for AUS in patients with a dBO remained unclear. Our retrospective analysis of one of the largest databases of male patients with continence surgery now renders a detailed assessment of the outcomes for this particularly burdened cohort possible.

## 2. Materials and Methods

Our multicenter cohort study included 473 male patients who received an AMS 800 sphincter between 2010 and 2012. We compared male patients who had received RT for locally advanced or relapsed prostate cancer only and patients with both RT and dBO (defined by SUI and recent surgery for mU/BN stenosis). We obtained ethics approval for the retrospective analysis. All data were captured and recorded by independent external investigators. Analysis covered the number of cases for each center, the type of incontinence surgery, the position, number and location of cuffs, and any prior surgery or irradiation with a potential effect on the continence status. Endpoints were defined as the need for revision due to urethral erosion, infection, mechanical complications or persistent incontinence and the recovery of continence (social continence 0–1 pad/24 h, respectively). The software used for statistical analysis was SPSS for Windows (Version 29.0). Contingency tables, Pearson chi square, Student’s *t*-test and Mann–Whitney U-Test were used for evaluation. We employed the Kaplan–Meier method to analyze the time to revision and calculated differences between the curves with the log rank test. Regression analysis of multivariate binary data included the variables RT only, 1x transurethral desobstruction, ≥2 transurethral desobstructions, diabetes mellitus, double cuff/single cuff (DC/SC), perineal/penoscrotal implantation, usage of 3.5 cm cuff, salvage AUS, and age </> 65 years.

## 3. Results

From the initial 473 patients, we identified 123 who had received pelvic RT prior to implantation of an AUS (no information on the exact time of the RT in 50 cases; 6 of 73 cases (4.9%) primary RT; 67 of 73 (91.8%) adjuvant/salvage RT). Of the 123 males, 62 (50.4%) required an additional desobstruction beforehand due to mU/BN stenosis. The interval from RT in this group was 86 months (vs. 74 months for RT only). The treatments used were: transurethral resection (TUR), incision, stent implantation, and open reconstruction (Table 1). A total of 33 cases (53.2%) required multiple procedures. A comparison of patients with RT only and patients with an additional dBO showed no differences in age, BMI, prevalence of diabetes mellitus, and type of radiation (Table 1). Likewise, the number and type of previous surgeries did not differ significantly between the two groups. Looking at the AUS implantation technique, we found a difference in the chosen cuff size (4.48 cm vs. 4.26 cm RT only, *p* = 0.03). In patients with a dBO, a transcorporal (TC) implantation technique was used in 24.4% of cases. SC/DC and perineal/penoscrotal were not significantly differently distributed between the groups (Table 1).

**Figure 1:**
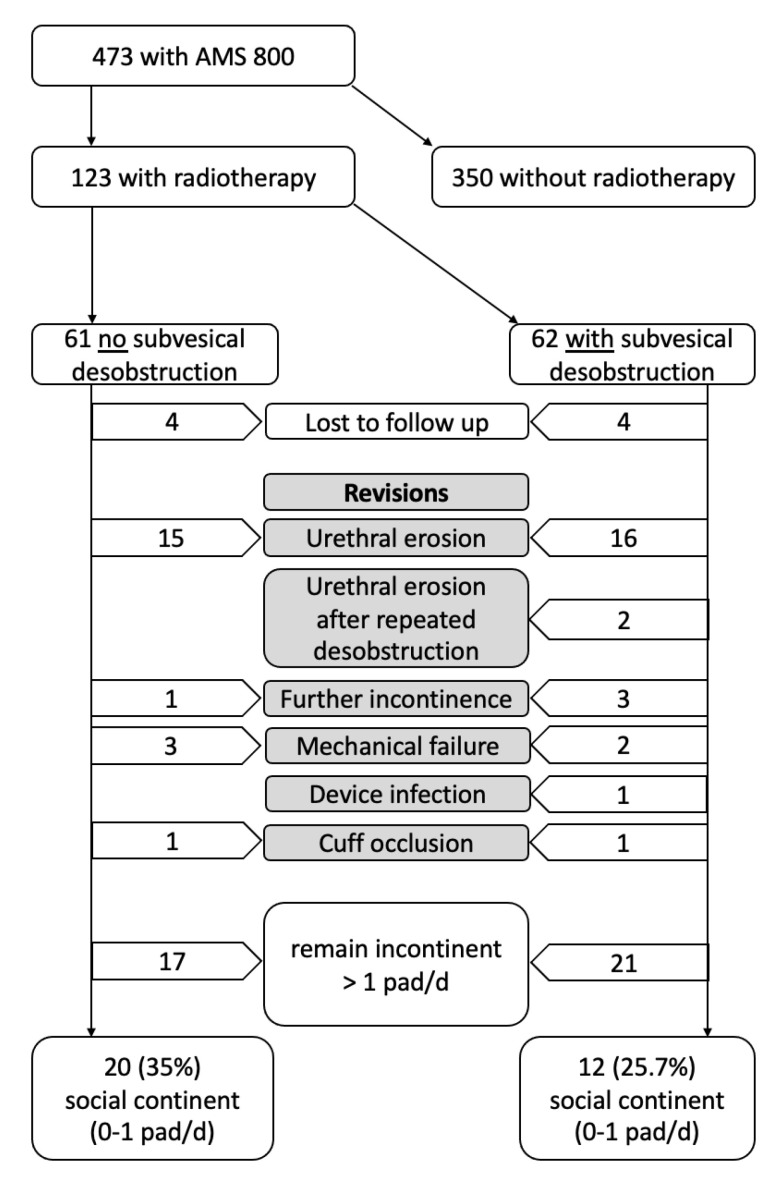
Flow diagram of our cohort with dropouts.

The median follow-up for our cohort was 21 months. Revision of the AUS was required significantly more often for patients with a dBO in 25/62 (43.1%) of cases (Figure 1, Table 2).

**Figure 2:**
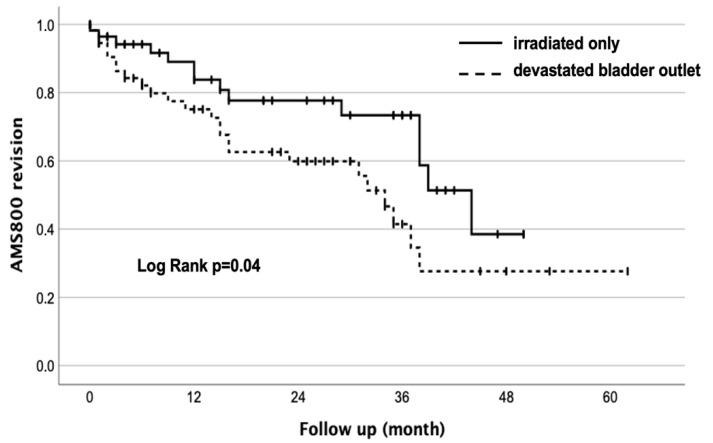
Kaplan-Meier analysis of revision needed—Irradiation only vs. irradiation and devastated bladder outlet.

Urethral erosion was the most common reason registered in 18 (31%) cases. In all males, the AUS was removed, and information about the method of urethral reconstruction (secondary healing vs. end-to-end urethroplasty) was not documented. Persistent/recurrent incontinence led to revision in three (5.2%) cases. Social continence tended to be achieved more frequently in patients without prior desobstruction in 20/61 (35%) cases vs. 12/62 (25.7%) (*p* = 0.08). In the Kaplan–Maier analysis, 50% of patients with RT alone required a revision after 37 months, while 50% of patients with a dBO required a revision after 26 months (log rank *p* = 0.04, Figure 2). A total of 12 of 20 (60%) males with RT and prior desobstruction needed revision when treated in low-volume centers (<10 AMS 800/year) compared to 13 of 38 (34%) in high-volume centers (≥10 AMS 800/year; *p* = 0.06).

Revision tended to be necessary in 7 of 23 (30.4%) cases, hence less frequently (*p* = 0.06) for patients who required transurethral desobstruction only once. If the stenosis recurred, revision became necessary in 13/24 (54%) cases. AUS explantation was performed on 5/7 (71.4%) men after open perineal urethroplasty. For patients with TC implantation, revision was necessary in 33.3% of cases and, for patients with standard surgical technique, in 41.4% of cases (*p* = 0.7). In 5/123 cases (4.0%), stenosis recurred after AMS 800 implantation. Transurethral bladder-neck/anastomosis incision was performed in two cases, in both of which urethral erosion occurred postoperatively.

The multivariate binary regression analysis of the need for revision included the following variables: High-volume centers (≥10 AMS/y), radiotherapy only, 1x transurethral desobstruction, ≥2 transurethral desobstructions, diabetes mellitus, DC/SC, perineal/penoscrotal, use of a 3.5 cm cuff, salvage AUS, and age </> 65 y. It showed that only patients with at least two desobstructions (HR 1.8, *p* = 0.028) were at an increased risk of revision, whereas patients treated in high-volume centers were at a lower risk (HR 0.27, *p* = 0.03).

## 4. Discussion

Patients with pelvic RT and status post RPx incur two risks: on the one hand, a risk of developing a stenosis of mU/BN, and, on the other, of developing SUI. Both entities are based on transformation processes in the irradiated tissue. Decreased perfusion, an inability to heal, and a fibrotization can lead directly to a stricture or damaging of the sphincter [6,12]. Indirectly, transurethral instrumentation with the necessary passage of a previously damaged membranous urethra can also lead to secondary stress incontinence. Our research group has now been able to demonstrate, for a large cohort of irradiated patients who received an AMS 800, that they run a high risk of needing revision if they also present with a dBO (additional stenosis of mU/BN and SUI). After little more than 2 years, a revision was already necessary in 50% of our patients after RPx, RT and previous transurethral desobstruction, with erosion being the preponderant underlying cause. Only 25.7% achieved the goal of social continence. We were able to show for the first time that recurrent mU/BN stenosis tended to be associated with a worse outcome (all revisions 54.0% vs. 30.4%). In our opinion, the term “devastated bladder outlet”, first mentioned by Riedmiller et al. [7], should also be applied to this group. With that, we augment the EAU guidelines [1], which discussed a worse outcome only for RT, penoscrotal approach, age, and interval RPx—continence surgery. Patients in our cohort who had open perineal reconstruction were at particular risk. In five of seven cases, the AMS 800 was removed because of cuff erosion. Sayedahmed et al. demonstrated a threefold increased risk of revision after prior urethroplasty in their study group, which excluded irradiated patients and in which only 26.7% of cases had prior RPx [13]. Earlier small series showed promising results of AMS 800 after urethroplasty but also excluded irradiated patients [14]. However, Mc Geady et al., in a more heterogeneous cohort that also comprised irradiated patients, found an 8.6-fold increased risk for their patients with open urethroplasty compared with patients without risk factors. They concluded that the blood supply from transection of the urethra and ligation of remaining bulbar vessels heightened this risk [15]. In their retrospective analysis of patients with a “fragile urethra”, Mann et al. also reported an earlier onset of erosion with status post open urethroplasty (HR 2.12) in addition to radiation (HR 2.36) [16]. Mann et al. used the term “fragile urethra” for men who had 1. status post radiation, 2. status post urethroplasty, or 3. status post a second AUS. However, in the Kaplan–Meier analysis, a comparatively worse outcome was confirmed only for the first two items, RT and open urethroplasty. Other study groups also reported no significantly higher revision rates for patients with a salvage AUS [11]. Still other study groups used the term “fragile urethra” in the context of an AUS implantation [17,18,19] but did not investigate the influence of the individual baseline variables. Hoy et al. added the cystoscopic factor in urethral atrophy to the definition [19]. Our results suggest that the definition of “fragile urethra” should be expanded by considering a status post recurrent urethral stenosis even without prior open reconstruction. It is still an open question whether patients with such a constellation should be offered AUS implantation at all. Alternative open reconstructive surgical procedures, such as bladder-neck closure, continent vesicostomy optionally combined with augmentation or urinary diversion with or without cystectomy, are currently being discussed [7]. However, the acceptance of these procedures at present can only be regarded as low. In the quest for strategies in the presence of a “fragile urethra”, the TC implantation technique is seeing increased use [18,20,21,22,23]. Initially in 2002, this procedure was described by Guralnick et al. in patients with distal reimplantation of an AMS 800 after erosion of a primary AUS [23]. However, it is also becoming increasingly common in primary applications. Redmond et al., and later Miller et al., described a significantly lower rate of major complications and operative revisions for the TC coupled with better continence outcomes in irradiated patients [18,22]. Le Lond also reached this conclusion but only included patients with a primary AMS 800 implantation [20]. Ortiz et al. investigated the localization of erosion in patients with the standard and the TC technique and found a higher erosion rate in TC patients (18.3% vs. 6.1%) but no difference in the localization of erosion [24]. Both groups showed ventral erosion in most cases (TC 66.7% vs. 79.5%), a localization where no greater supporting tissue arises from using the TC technique. However, as in Ortiz et al., most studies comparing the standard procedure and TC suffer from a considerable selection bias: The decision about which surgical procedure to use is made intraoperatively or on the basis of anamnestic factors (“fragile urethra”). Consequently, the TC group is characterized by a larger proportion of old, irradiated, and urethrally preoperated patients. Our dataset precludes us from making a clear statement on this issue given that it only includes 10 patients who underwent TC implantation. However, due to the growing body of evidence and in view of the high erosion rate when using a 3.5 cm cuff [25], the TC technique is now regularly used in cases involving a previously damaged, atrophied urethra. This might explain our tendentially lower revision rate when patients with prior desobstruction were operated in high-volume centers. Males with a fragile urethra need an experienced surgeon with a wide armamentarium of surgical techniques including TC implantation.

In some cases, the use of adjustable sling systems such as the Adjustable TransObturator Male System (ATOMS) can also be offered as an option. That said, the retrospective analysis by Ullate et al. indicates a significantly reduced effectiveness of this system in men with a dBO [26]. Therefore, ATOMs implantation should only be utilized in patients with a good residual sphincter function. In addition to spontaneous urethral erosion, iatrogenic injury of the urethra during transurethral manipulations (catheterization, transurethral surgery) is common, constituting the main cause of erosion, especially in cases of late erosion >2 years postoperatively [27]. In two of our devastated bladder outlet patients, transurethral desobstruction was performed for recurrent stenosis of the bladder neck. In both cases, erosion occurred postoperatively. Desobstruction should therefore not be indicated lightly and surgery performed only in cases of pronounced symptoms (impending urinary retention). New surgical techniques featuring a pediatric cystoscope or semirigid ureteroscope and making a laser incision avoid the application of leverage forces on the cuff-bearing urethra and prevent coagulation necrosis. Weissbart et al. and Ramirez et al. observed no major complications after using these surgical techniques in several patients with AUS [28,29]. Unfortunately, information on the time interval between the last desobstruction and AUS implantation was missing in our retrospective analysis. Well-founded statements about the optimal time interval are therefore not possible based on our data. Myers et al. discussed that the decision to implant AUS should best be made within the scope of an endoscopic control about 3 months after desobstruction [30]. If the reopened stenosis heals adequately, reimplantation can take place. Necrosis and a narrow recurrence should result in a longer wait or other treatment strategies.

## 5. Conclusions

The term “fragile urethra” is used for AUS candidates with (1) a status post radiotherapy condition, (2) a urethral erosion secondary to a previous AUS implantation, or (3) a status post open urethroplasty. Our findings of irradiated patients with a dBO, i.e., the combination of SUI and stenosis of the mU/BN, versus patients who were irradiated only, argue for broadening the term “fragile urethra” to include this entity. A significantly higher revision rate and a low proportion of socially continent patients require an adaptation of surgical strategies for this challenging cohort. One possibility is the increasingly used TC technique, whose protective effect, however, has yet to be proven by prospective randomized studies. We assume that the AMS 800 is still an option for patients with a devastated bladder outlet; however, other options, such as urinary diversion or bladder-neck closure with vesicostomy, can also be considered.

## Figures and Tables

**Table 1:** Baseline characteristics of patients with radiotherapy (RT) with/without previous desobstruction of membranous urethra/bladder neck (mU/BN).

	RT Only	RT and Surgery for mU/BN Stenosis	
Pat. (*n*)	61	62	
Age (mean)	75.5 (± 6.8)	74.4 (± 6.0)	*p* = 0.3 *
BMI (mean)	28.6 (± 4.9)	28.0 (± 4.2)	*p* = 0.4 *
Diab. mel. (*n*)	16 (26.2%)	10 (16.1%)	*p* = 0.17 **
Brachytherapy (*n*)	1 (1.6%)	2 (3.2%)	*p* = 1 **
External irradiation	60 (98.4%)	60 (96.8%)	*p* = 1 **
**Prior incontinence surgery**	19 (31.1%)	19 (30.6%)	*p* = 1 **
>1 prior surgery	6 (9.8%)	3 (4.8%)	*p* = 0.3 **
Bulking agent	4 (6.7%)	4 (6.5%)	*p* = 1 **
Fixed sling	8 (13.1%)	6 (9.7%)	*p* = 0.6 **
Adjustable sling	4 (6.7%)	3 (4.8%)	*p* = 0.7 **
proAct	2 (3.2%)	0	*p* = 0.2 **
AUS	6 (9.8%)	7 (11.3%)	*p* = 1 **
	**RT Only**	**RT and Surgery for** **mU/BN Stenosis**	
**Prior surgery for mU/BN Stenosis**			
*n* > 1		33 (53.2%)	
UI		29 (46.8%)	
Bladder-neck resection/incision		32 (51.6%)	
Stent		5 (8.1%)	
Open urethroplasty		7 (11.3%)	
**AUS surgery**			
SC	26 (42.6%)	30 (48.4%)	*p* = 0.6 **
DC	35 (57.4%)	32 (51.6%)	*p* = 0.6 **
Perineal	44 (72.1%)	50 (80.6%)	*p* = 0.3 **
Penoscrotal	17 (27.9%	12 (19.4%)	*p* = 0.3 **
TC	unknown	10 of 41 (24.4%)	
Cuff size (cm, mean)	4.26	4.48	*p* = 0.03 ***
Cuff 3.5 cm	4 (6.7%)	7 (11.3%)	*p* = 0.4 ***

* *t*-Test, ** Pearson Chi Square, *** Mann–Whitney U.

**Table 2:** Outcome of males with radiotherapy (RT) only vs. RT and previous desobstruction of membranous urethra/bladder neck (mU/BN).

	RT Only	RT and Surgery for mU/BN Stenosis	
Lost to follow-up	4 (6.6%)	4 (6.5%)	
Follow-up (mean month)	20.5	21.5	
**Revisions**	15 (26.3%)	25 (43.1%)	*p* = 0.05 *
Erosion	11 (17.5%)	18 (31.0%)	*p* = 0.15 *
Incontinence	1 (1.8%)	3 (5.2%)	*p* = 0.3 *
Mechanical failure	3 (5.3%)	2 (3.4%)	*p* = 0.6 *
Infection	0	1 (1.7%)	*p* = 0.3 *
Cuff occlusion	1 (1.8%)	1 (1.7%)	*p* = 1 *
**Functional outcome**			
Social continence (0–1 pad/24 h)	20 (35.0%)	12 (25.7%)	*p* = 0.08 *

* Pearson chi square.

## Data Availability

The data are not made freely available for privacy reasons but can be requested from the author by email: fabian.queissert@ukmuenster.de.
